# Supplementary figures and images for: Mitochondrial Genotoxicity of Hepatitis C Treatment among People Who Inject Drugs
Source: J Clin Med. 2021 Oct 20;10(21):4824. doi: 10.3390/jcm10214824 (PMC8584601; doi:10.3390/jcm10214824)

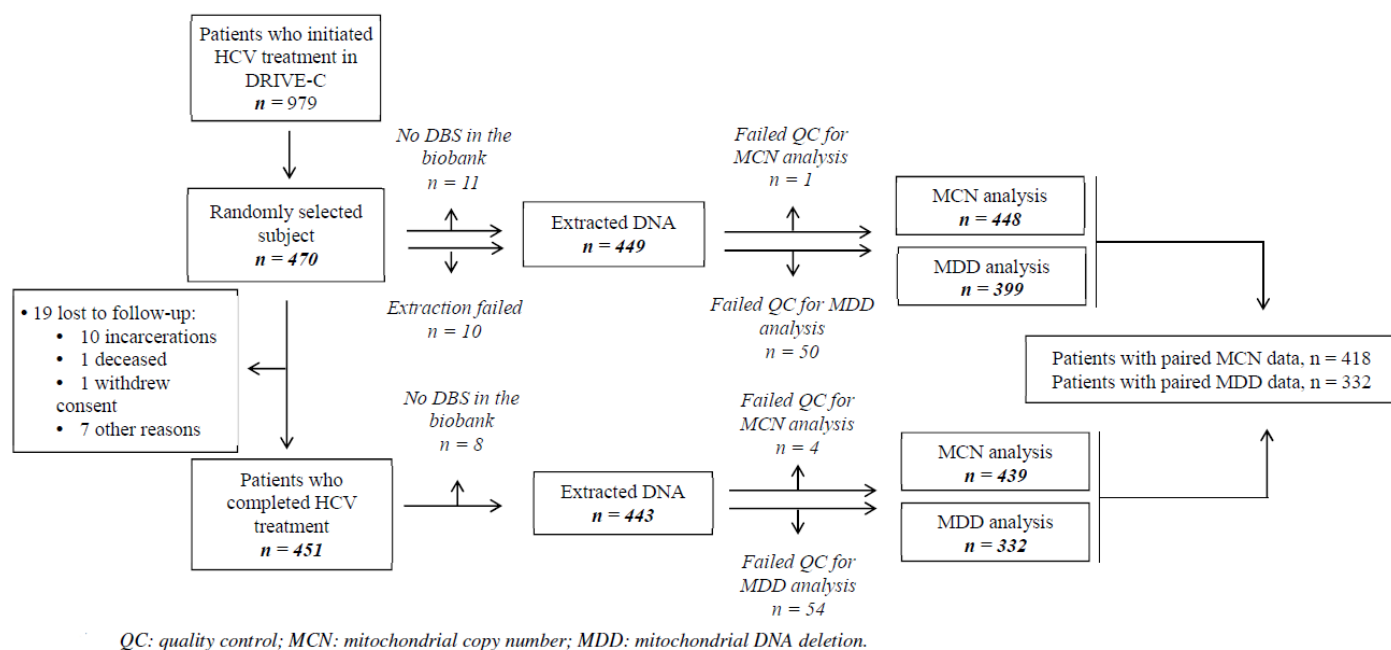

**Supplementary Figure S1.** Sample flow chart.

Supplement: Supplementary file 1 [file jcm-10-04824-s001.zip › jcm-1429116-supplementary.pdf]
